# Sellar hemangiopericytoma masquerading as pituitary adenoma: an overlooked intriguing case study unveiling a rare surgical conundrum

**DOI:** 10.3389/fsurg.2024.1359787

**Published:** 2024-05-22

**Authors:** Kaveh Ebrahimzadeh, Mohammad Mirahmadi Eraghi, Mohammad Ansari, Adam A. Dmytriw

**Affiliations:** ^1^ Skull Base Research Center, Loghman Hakim Hospital, Shahid Beheshti University of Medical Sciences, Tehran, Iran; ^2^ Brain and Spinal Cord Injury Research Center, Neuroscience Institute, Tehran University of Medical Sciences, Tehran, Iran; ^3^ Student Research Committee, School of Medicine, Islamic Azad University, Qeshm International Branch, Qeshm, Iran

**Keywords:** hemangiopericytoma, sellar hemangiopericytoma, pituitary adenoma, sellar tumor, endoscopic trans-sphenoidal surgery

## Abstract

Hemangiopericytoma (HPC) constitutes less than 1% of all primary central nervous system tumors. It is a vascular neoplasm with potential malignancy that, in rare instances, manifests as a primary lesion within the brain. Typically, it originates from the meninges. Here, we describe an exceptionally uncommon sellar region solitary fibrous tumor/hemangiopericytoma (SFT/HPC) that mimicked a nonfunctional pituitary adenoma.

**Introduction and importance:**

**Case presentation:**

A 54-year-old male was referred to our hospital due to progressive blurred vision in the left eye over the past year. A homogeneous iso-dense extra-axial intrasellar round mass with extension into the suprasellar region, mainly on the left side, along with bony erosion and osteolysis around the sellar region, was observed on a brain computed tomography (CT) scan. Brain magnetic resonance imaging (MRI) revealed a well-defined 251,713 mm mass with iso-signal on T1-weighted images and hypersignal on T2-weighted images, originating from the pituitary gland within the sella turcica. The mass avidly enhanced following Gadolinium injection and adhered to both carotid arteries without vascular compression or invasion. It extended to the suprasellar cistern and compressed the optic chiasm. The diagnosis was nonfunctional pituitary macroadenoma, leading to the decision for Endoscopic Trans-Sphenoidal Surgery (ETSS). A non-sustainable, soft, grayish mass was grossly and totally resected during the operation. Subsequently, there was a significant improvement in visual acuity during the early postoperative period. Histopathologic examination confirmed hemangiopericytoma (WHO grade II).

**Conclusion:**

Due to its malignant nature, hemangiopericytoma should be included in the differential diagnosis of a sellar mass, both from a clinical and morphological perspective.

## Highlights

• A vascular neoplasm known as hemangiopericytoma has the potential for malignancy and occasionally appears as a primary lesion within the brain.
• In terms of both ophthalmological manifestations and endocrine dysfunction, intracranial hemangiopericytoma can simulate the characteristics of a pituitary tumor.
• The recommended approach for treating hemangiopericytoma involves surgical removal followed by additional radiation therapy.

## Introduction

The rare tumor known as solitary fibrous tumor (SFT)/hemangiopericytoma (HPC) was initially documented by Begg and Garret in 1954. It originates from the capillary walls and has historically been believed to have the potential to develop anywhere in the body, although occurrences in the sellar region are infrequent (1, 2). In 1942, Stout and Murray coined the term “hemangiopericytoma” to describe a vascular tumor arising from Zimmermann's pericytes, which are altered smooth muscle cells located within the capillary walls, forming endothelial tubes and sprouts (3). Since then, HPCs have undergone several changes in nomenclature. Ultimately, extracranial HPCs have been reclassified under the category of “solitary fibrous tumors,” while neuropathologists continue to use the term “hemangiopericytoma” (4). SFTs or HPCs make up less than 1% of all primary central nervous system (CNS) tumors. Predominantly situated in the dura, these tumors are believed to originate from meningeal capillary pericytes, placing them in locations similar to meningiomas. However, SFTs or HPCs in the sellar and suprasellar regions are even more uncommon (4, 5). We present an exceedingly rare case of a sellar region SFT/HPC that resembles a nonfunctional pituitary adenoma.

## Case presentation

A 54-year-old male was referred to our hospital due to progressive blurred vision in the left eye over the past year. He reported a gradual loss of visual acuity in the left eye and experienced daily throbbing biparietal headaches, relieved by analgesics. Additionally, he mentioned polyuria, polydipsia, and a loss of libido dating back approximately one year. His medical history included rheumatoid arthritis, hypertension, and diabetes mellitus induced by glucocorticoids. He was recently taking losartan (50 mg daily) and prednisolone (5 mg daily). There was no notable family history.

**Figure 1:**
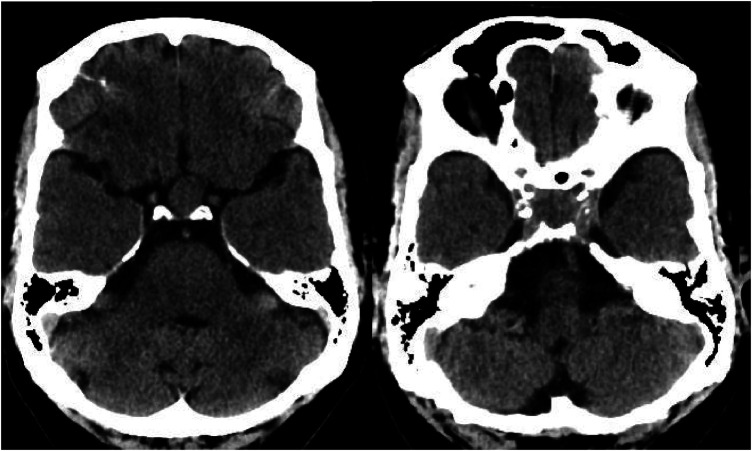
Brain CT scan without contrast, axial view. Intrasellar mass with extension to suprasellar cistern and adjacent bone erosion.

**Figure 2:**
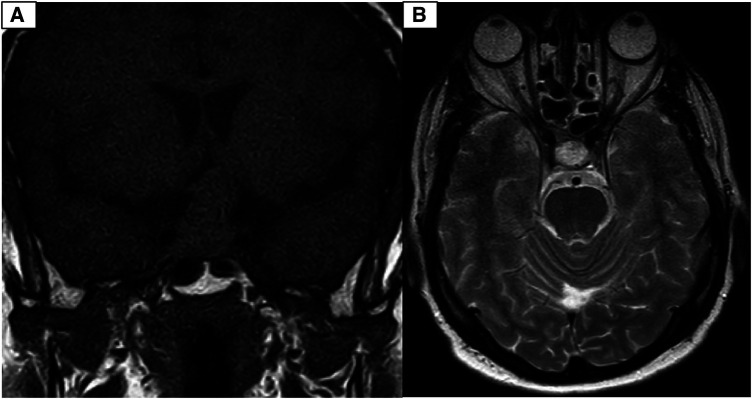
(**A**) Coronal view of T1-weighed brain MRI without Gadolinium injection. Well defined sellar mass with extension to suprasellar cistern. (**B**) Axial view of T2-weighed brain MRI without Gadolinium injection. Well defined sellar mass with extension to suprasellar cistern and adherent to both carotid arteries.

**Figure 3:**
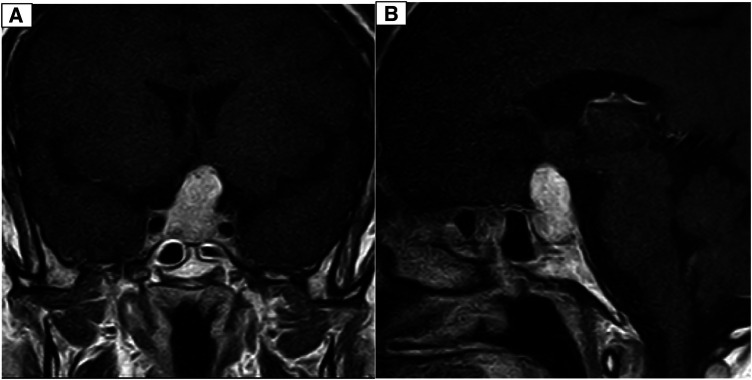
(**A**) Coronal T1-weighted brain MRI with Gadolinium contrast injection depicting a distinctive avidly enhanced sellar mass extending to the suprasellar cistern and adhering to both carotid arteries. (**B**) Sagittal T1-weighted brain MRI with Gadolinium contrast injection reveals a well-defined, prominently enhanced sellar mass extending into the suprasellar cistern and adherence to both carotid arteries.

During the physical examination, bilateral visual acuity loss was observed (right eye: 5 meters counting fingers, left eye: 0.5 meters counting fingers). Ocular movements and other cranial nerves were normal, with no abnormalities detected in the peripheral nervous system and other neurological examinations. A brain computed tomography (CT) scan revealed a homogeneous iso-dense extra-axial intrasellar round mass extending to the suprasellar region, predominantly on the left side, with bony erosion and osteolysis around the sellar region (Figure 1). Brain magnetic resonance imaging (MRI) showed a well-defined mass with iso-signal on T1-weighted images and hypersignal on T2-weighted images, originating from the pituitary gland within the sella turcica. The mass avidly enhanced following Gadolinium injection and was adherent to both carotid arteries without vascular compression or invasion, extending to the suprasellar cistern and compressing the optic chiasm (Figures 2, 3). Hypothalamic-pituitary-adrenal axis evaluation revealed no hormonal abnormalities.

**Figure 4:**
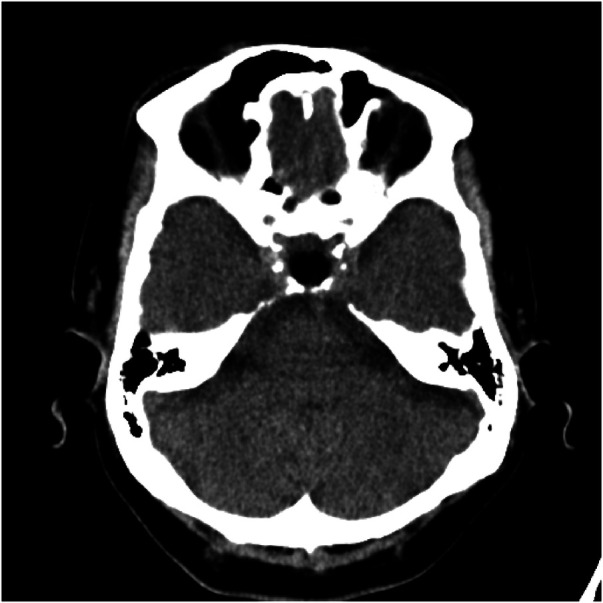
A post-operative brain CT scan, conducted without contrast injection, depicted the resection of the sellar and suprasellar mass, along with the implantation of abdominal subcutaneous fat tissue.

The diagnosis was a non-functional pituitary macroadenoma, leading to the decision for endoscopic trans-sphenoidal surgery (ETSS). Intraoperatively, a soft grayish non-sustainable mass was grossly resected. There was a remarkable improvement in visual acuity during the early postoperative period (right eye: 5 meters counting fingers, left eye: 5 meters counting fingers), and a postoperative brain CT scan showed no residual mass (Figure 4). During the operation, we encountered a soft grayish sellar mass with suprasellar extension and peripheral bone invasion and erosion.

The tumor had extended to the floor of the 3rd ventricle. Following surgery, the patient experienced meningitis and cerebrospinal fluid (CSF) leakage. Treatment included administration of intravenous antibiotics and acetazolamide. After completing a 21-day regimen, there was no CSF leak detected. Despite the tumor's lateral extension beyond the carotid arteries, into the middle fossa, and temporal region, which poses challenges for the endoscopic trans-nasal approach, the patient opted for ETSS due to the presence of approximately midline suprasellar extension without lateral spread.

**Figure 5:**
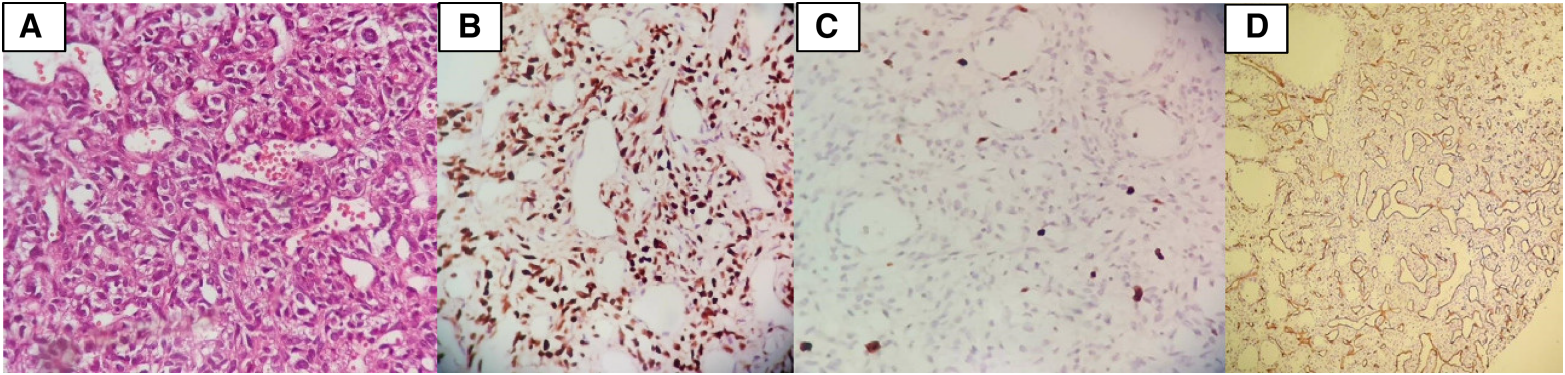
(**A**) Histopathologic examination revealed a hypervascular neoplasm of branching vessels surrounded by bland-looking pericytes with oval to spindle nuclei without evidence of anaplasia, necrosis, or hemorrhage. (**B**) IHC stain positive for STAT6 in tumoral cells. (**C**) IHC stain demonstrates that Ki67 is low. (**D**) IHC stain for CD34 highlights vascular proliferation.

Histopathologic examination revealed a hypervascular neoplasm of branching vessels surrounded by bland-looking pericytes with oval to spindle nuclei without evidence of anaplasia, necrosis, or hemorrhage. Immunohistochemistry staining was positive for STAT 6 in tumoral cells, while Ki67 was low, and CD-34 highlighted vascular proliferation—all consistent with HPC [World Health Organization (WHO) grade II] (Figure 5).

**Figure 6:**
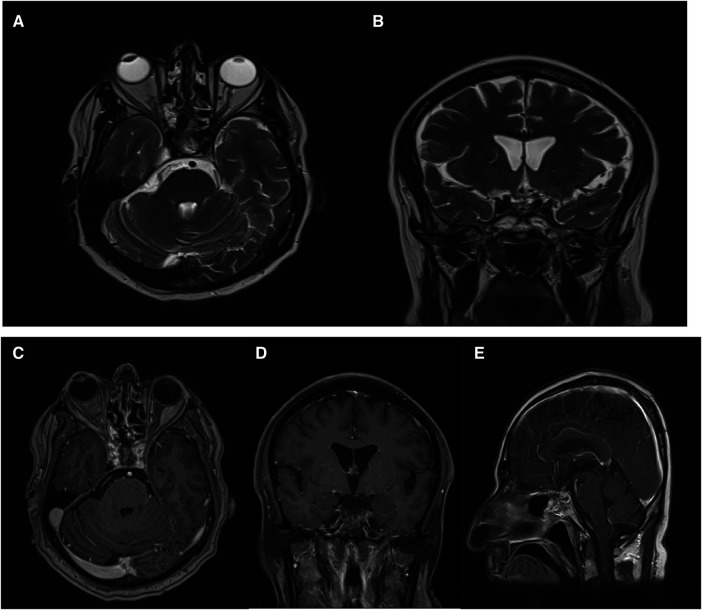
Post-operative brain MRI revealed no evidence of residual mass. Axial (**A**), coronal (**B**), axial with Gadolinium injection (**C**), sagittal with Gadolinium injection (**E**).

**Figure 7:**
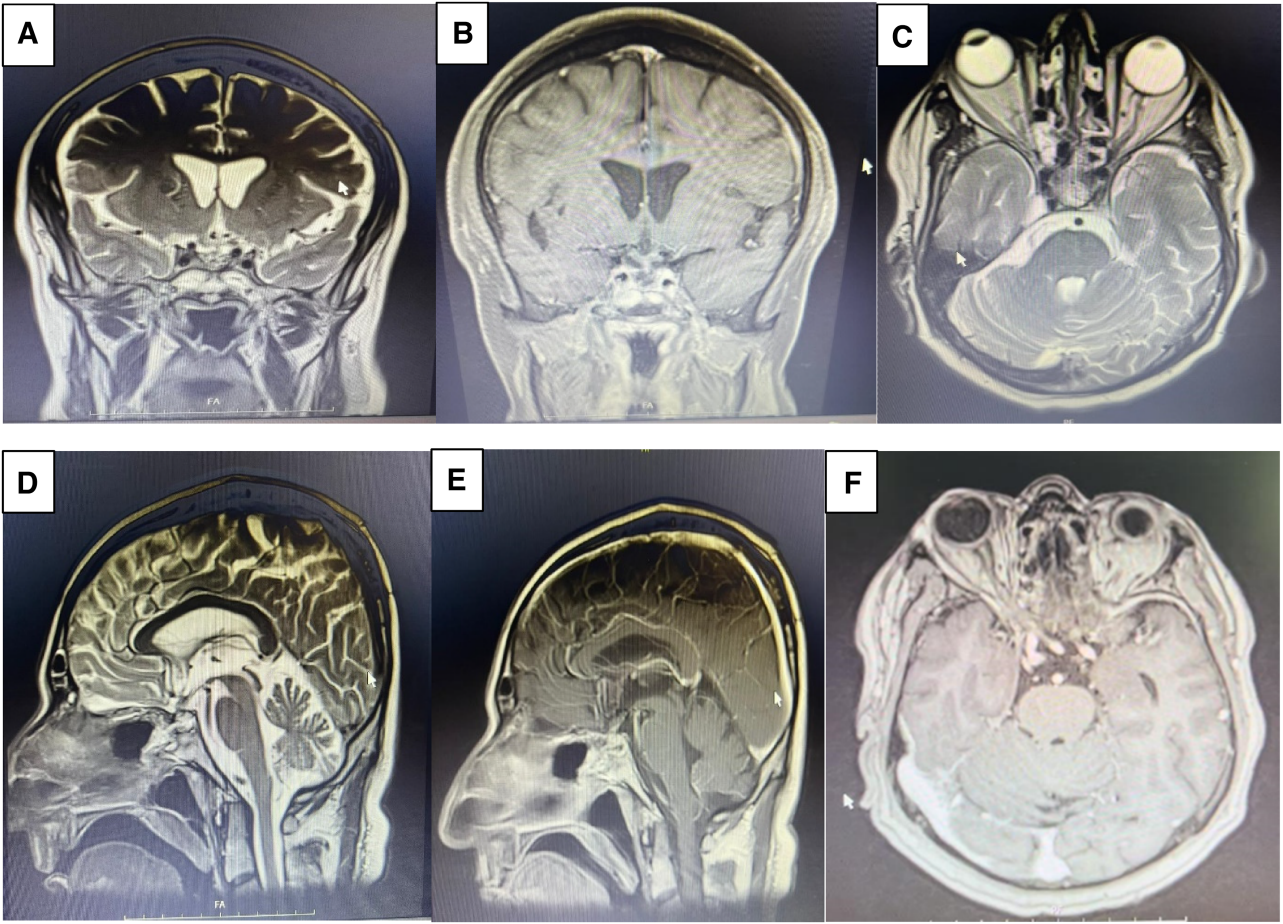
Post op MRI on month 2 demonstrates gross total resection (GTR) of the sellar mass with suprasellar extension. Coronal view of T2 weighed (**A**), coronal view of T1 weighted following Gadolinium injection (**B**), axial view of T2 weighed (**C**), sagittal view of T2 weighted (**D**), sagittal view of T1 weighted Gadolinium injection (**E**), axial view of T1 weighted following Gadolinium injection (**F**).

The patient had a history of ETSS, with observed postoperative changes in the sella. No abnormal enhancement affecting the brain parenchyma or meninges was detected. Both supra and infratentorial structures appeared grossly normal (Figures 6, 7).

## Discussion

HPCs and SFTs represent uncommon primary intracranial tumors. HPC constitutes only 1% of all intracranial tumors (6, 7). Approximately 15% of SFTs exhibit malignant biological behavior, while the majority display benign and mesenchymal characteristics (8). Recent investigations have identified a novel fusion gene involving Nab2 and STAT6 in samples of SFT and HPC. This discovery has led to the reclassification of these tumors into a new category known as SFT/HPC in the WHO classification of CNS tumors in 2016 (4). Despite several published reports detailing the pathological and clinical features, as well as survival outcomes of SFT/HPC, more comprehensive clinical data for this newly defined entity is still needed (9). Table 1 outlines the prominent characteristics of the previously reported sellar HPC mimicking pituitary adenoma.

**Table 1:** Literature review of the previous seller HPC mimicking pituitary adenoma.

Author	Gender/age	Location/diameter	Presentation	Hormonal irregularities	Pathology findings	Radiographic findings	Surgical management and intraoperative findings	Radiation therapy	Adverse event at follow up
Ghanchi et al. (2)	Male/12	Sellar mass with suprasellar extension	Headaches, visual impairmen, bitemporal hemianopsia t Polydipsia without diabetes insipidus	None	Staghorn appearance with additional positivity for CD34 and signal transducer and activator of transcription 6, confirming solitary fibrous tumor/ hemangiopericytoma, World Health Organization (WHO) Grade II	MRI: sellar lesion with suprasellar extension exhibiting homogenous enhancement	Transnasal transsphenoidal craniotomy Firm and fibrous lesion. Complete resection was not feasible Second surgery at month 3 of follow up: a left-sided orbitozygomatic craniotomy. Tumor was resected in a piecemeal fashion with an ultrasonic aspirator. Microsurgical techniques were used to dissect the tumor capsule Gross total resection was achived in the second surgery	No	On 3-month follow-up, progressive and enlarging lesion Worsening vision in the left eye, Second surgery: diabetes insipidus requiring desmopressin and transient loss of vision in the left eye
Juco et al. (10)	18/Female	Sellar mass with suprasellar extension	Unsteady gait visual disturbances in the left eye left temporal hemianopia Right superior quadrantanopia	None	Accumulation of basement membrane substance within the intercellular stroma. Ultrastructural characteristics of the tumor cells: relatively lucent nuclei with ropey nucleoli, aggregate of intermediate filaments and clusters of glycogen-β particles		Transnasal transsphenoidal Adense, rubbery, highly vascular gray semisolid tumor was resected. The normal pituitary gland was preserved, which was found compressed against the right posterolateral wall of the sella turcica	Yes	Early post op: poor restless sleep Last follow up: small incidental collection of blood vessels in the left occipital area, consistent with an arteriovenous malformation.
Kanda et al. (11)	60/Female	Sellar	Right visual disturbance and double vision incomplete bitemporal hemianopia	None	Perivascular proliferation of the cells with low differentiation and were spindle-shaped, with a narrow cytoplasm and a large nuclear-cytoplasmic ratio. Second operation: irregular nuclei were closely adjacent. Intermediate filaments and developed endoplasmic reticula	Isointensity on T1-, iso-intensity, including partial high intensity on T2-weighted MR images, and strong enhancement by Gd-DTPA. Right carotid angiography:opening of the carotid siphon and tumor staining fed by the branches of meningohypophyseal trunk.	First surgery: transsphenoidal approach Second surgery tumor 2 months later: right frontal craniotomy Optic nerve decompression without total removal of the tumor.	Yes	Early postoperation: Persistent bitemporal hemianopia
Morrison et al. (12)	35/Female		Patchy visual loss in the left eye Frontal headache Incomplete bitemporal field loss	Elevated serum prolactin(2,568 U/L)	Homogenous, highly cellular tumor with spindle-shaped cells having scanty eosinophilic cytoplasm. Vascular channels and sinusoids lined by a flattened endothelium	MRI: contrast-enhancing soft tissue mass arising from the pituitary fossa, with apparent compression of the chiasm and involving the right cavernous sinus	Transphenoidal resection of the tumor, which was soft, pale, and vascula	Yes	Early postoperative: Residual tumor

Extracranial SFT/HPCs have undergone reclassification within the spectrum of SFTs, while neuropathologists still commonly employ the term HPC (Table 2). Both entities exhibit the 12q3 inversion and fusion of the NGFI-A-binding protein 2 (NAB2) and STAT6 genes, with observable nuclear expression of STAT6 on immunohistochemistry (13). In the 2016 WHO classification of CNS tumors, these two tumors were collectively designated as a single entity, acknowledging their shared inversions at 12q13, resulting in the fusion of NAB2–STAT6 genes and subsequent nuclear expression of STAT6 (14).

**Table 2:** The most common differential diagnosis of sellar masses and therapeutic approach (18, 36, 37).

Differentia diagnosis	Radiologic features	Clinical management
Pituitary adenoma	Isointense on T1 and T2 heterogeneous enhancement	Surgery Radiation
Craniopharingioma	Solid/cystic	Surgery: cystic aspiration ±Radiation
Meningioma	Dural tail sign	Observation depending on the size, growth, and age Surgery ±Radiation
Astrocytoma/glioma	Large cystic lesion with brightly enhancing mural nodule or heterrogenous enhancement based on WHO grade	Surgery ±Radiation
Miscellaneous		
Aneurysms	In areas of slower turbulent flow: Flow void and heterogeneous increased signal intensity	Surgical clipping vs. endovascular coiling
Germ cell tumors	Soft tissue mass, ovoid or lobulated heterogeneous enhancement DWI restricting mass	Radiation Chemotherapy
Hypothalamic glioma	T1 enlargment iso to hypointense copared to contralateral side T2 hyperintense	Chemotrapy Surgery Radiation
Rathke's cleft cyst	Intracystic micro nodule, best seen on T2-weighted images	Observation Surgery
Hamartoma	Soft tissue iso-attenuating to grey matter, lack of calcification or contrast enhancement	Precocious puberty medical Epilepsy-surgery
Chordoma	Lytic lesion of the clivus, with intra-tumoral septa	Surgery Radiation
Lymphoma	Isointese on T1 and T2 contrast enhancement	Chemotrapy Radiation
Hemangiopericytoma	Heterogeneous, isointense mass with gray matter on T1-weighted, slightly hyperintense on T2-weighted sequences, as well as signal vessel voids.	Surgery Radiation

STAT6, or Signal Transducer and Activator of Transcription 6, plays a pivotal role in a myriad of cellular processes, encompassing growth, survival, differentiation, and immune responses. Recent investigations have unveiled a distinctive molecular profile linked to hemangiopericytoma, characterized by recurring NAB2-STAT6 gene fusions. This fusion event leads to the perpetual activation of the STAT6 signaling pathway, speculated to augment the oncogenic potential of HPC. Several studies indicated that robust nuclear STAT6 expression, as identified through immunohistochemistry, serves as a highly sensitive and specific marker for HPC. Furthermore, the presence of the NAB2-STAT6 fusion gene has been correlated with a more favorable prognosis in HPC patients (15).

While the precise origin of SFT or HPC remains uncertain, there is consensus on its mesenchymal nature characterized by HPC-like features, such as a monotonous cell population, varying cellularity, and the presence of branching blood vessels (16). Ultrastructurally, both SFT and HPC exhibit diverse degrees of pericytic, fibroblastic, or myofibroblastic differentiation. Gengler and Guillou clarified that, excluding myopericytoma, infantile myopericytosis, and HPC of the sinonasal tract, all HPCs lacking pericytic differentiation are considered variants of SFT, resembling a cellular form of SFT within a morphological continuum (16). Tumors characterized by lower cellularity and high collagen content are designated as grade I (formerly SFT), whereas those with increased cellularity, reduced collagen, and characteristic staghorn vasculature are categorized as grade II (formerly HPC). Grade III encompasses HPC or SFT with five or more mitoses per 10 high-power fields, inclusive of both anaplastic HPC and malignant SFT types (4).

Sellar SFTs or HPCs are frequently misidentified as pituitary adenomas and meningiomas (17). Visual disturbance followed by headaches is the predominant presenting symptom of sellar SFT or HPC. Like other suprasellar tumors, these growths often compress the pituitary gland and stalk, resulting in common symptoms of pituitary dysfunction (10, 17).

Imaging characteristics of HPC encompass several aspects: a broad-based attachment to the dura, absence of calcifications and hyperostosis, multilobulated tumor morphology, heterogeneously hyperdense patterns with focal areas of hypodensity on unenhanced brain CT, varied enhancement patterns on enhanced brain CT (homogeneous or heterogeneous), and an isointense signal compared to cortical gray matter on T1- and T2-weighted brain MRI. Additionally, they exhibit heterogeneous enhancement on Gadolinium-enhanced T1-weighted brain MRI (18). Intracranial SFT's CT features closely resemble those of extracranial SFT, displaying isoattenuation and marked enhancement following intravenous iodinated contrast injection. Brain MRI highlights SFT's extra-axial, multilobulated nature with heterogeneous signal intensity on T2-weighted images, hypointense T2 areas exhibiting robust enhancement after gadolinium administration, while adjacent meninges remain unenhanced (19). Table 2 outlines the most common differentiation diagnosis of sellar masses and therapeutic approach.

For intraspinal SFT/HPC, a comprehensive en-bloc surgical resection is advisable to mitigate postoperative recurrence or regrowth (17). However, due to the fibrous, hypervascular, and invasive nature of these tumors, partial resection is the more commonly employed surgical management. While total removal has been achieved in certain instances, some cases have reported new-onset visual impairment and pituitary dysfunction postoperatively (20, 21).

Divergent opinions exist among researchers regarding the optimal strategy for HPC management. Some advocate for complete surgical resection followed by postoperative radiotherapy to the tumor bed as the most effective approach (22, 23). A perspective suggests that external irradiation of the tumor bed after surgery may delay recurrence (24). Postoperative radiotherapy has been traditionally considered a primary treatment for HPC, although a previous study indicated that it did not significantly enhance local control and overall survival. Notably, the local control rates were comparable between intensity-modulated radiotherapy (IMRT) and stereotactic radiosurgery (SRS), despite IMRT having a substantially higher biological dose (25).

In our management, since HPC was not initially suspected as the etiology, and both intraoperative findings and postoperative MRI results confirmed gross total resection (GTR), we followed the protocol for pituitary adenoma, considering it the most likely diagnosis. However, upon receiving the pathology report confirming the diagnosis of HPC, the patient was referred to a senior oncologist for further advice regarding the potential need for radiotherapy.

Several studies indicate that postoperative radiotherapy following subtotal resection (STR) can enhance overall survival (OS) and recurrence-free survival (RFS) compared to STR alone (26–28). Additionally, postoperative radiotherapy following GTR has been associated with prolonged OS (6, 23, 29, 30) or improved local control (31, 32). In contrast, some authors report that postoperative radiotherapy after GTR has no significant impact on survival (26, 33) or should only be considered for recurrent cases (34, 35). Gou et al. (9) found that different postoperative radiotherapy (PORT) strategies significantly influenced disease-free survival (DFS). Patients receiving postoperative radiotherapy via IMRT had a median DFS of 8.33 years, a statistically significant difference compared to those undergoing postoperative radiotherapy via SRS. Multivariate analysis revealed that postoperative radiotherapy had no predictive effect on survival compared to surgery alone, but when compared to other treatments, both postoperative radiotherapy and surgery were beneficial for DFS.

In a series report involving 29 patients with intracranial adult HPC, the 5-, 10-, and 15-year overall survival rates were recorded at 85%, 68%, and 43%, respectively, for individuals undergoing this particular treatment regimen (27). Another series, encompassing 43 patients, reported 1-, 5-, and 10-year overall survival rates of 100%, 94.4%, and 72.2%, respectively (23). The outcomes are intricately linked to the presence of metastatic disease and the effectiveness of local control, with GTR being advocated due to its association with a 5-year local disease control rate of 84%, significantly higher than the 38% observed with STR (33).

## Conclusions

Although intracranial HPC is a rare entity, its clinical presentation can mimic a pituitary tumor both ophthalmologically and in terms of endocrine dysfunction. However, the prognosis is considerably more severe. The treatment approach is markedly distinct, necessitating extended follow-up. Histological confirmation remains crucial for optimal management, even when clinical features appear indicative of the diagnosis.

## Data Availability

The raw data supporting the conclusions of this article will be made available by the authors, without undue reservation.
